# ROI-Based On-Board Compression for Hyperspectral Remote Sensing Images on GPU

**DOI:** 10.3390/s17051160

**Published:** 2017-05-19

**Authors:** Rossella Giordano, Pietro Guccione

**Affiliations:** Department of Electrical and Information Engineering, Politecnico di Bari, 70125 Bari, Italy; giordanorossella88@gmail.com

**Keywords:** hyperspectral imaging, region-of-interest, clustering, on-board compression, PCA, GPU

## Abstract

In recent years, hyperspectral sensors for Earth remote sensing have become very popular. Such systems are able to provide the user with images having both spectral and spatial information. The current hyperspectral spaceborne sensors are able to capture large areas with increased spatial and spectral resolution. For this reason, the volume of acquired data needs to be reduced on board in order to avoid a low orbital duty cycle due to limited storage space. Recently, literature has focused the attention on efficient ways for on-board data compression. This topic is a challenging task due to the difficult environment (outer space) and due to the limited time, power and computing resources. Often, the hardware properties of Graphic Processing Units (GPU) have been adopted to reduce the processing time using parallel computing. The current work proposes a framework for on-board operation on a GPU, using NVIDIA’s CUDA (Compute Unified Device Architecture) architecture. The algorithm aims at performing on-board compression using the target’s related strategy. In detail, the main operations are: the automatic recognition of land cover types or detection of events in near real time in regions of interest (this is a user related choice) with an unsupervised classifier; the compression of specific regions with space-variant different bit rates including Principal Component Analysis (PCA), wavelet and arithmetic coding; and data volume management to the Ground Station. Experiments are provided using a real dataset taken from an AVIRIS (Airborne Visible/Infrared Imaging Spectrometer) airborne sensor in a harbor area.

## 1. Introduction

A hyperspectral dataset is a data cube acquired by a multi band camera that is composed of a number of images equal to the number of bands, with the purpose of detecting, identifying and classifying objects. Hyperspectral images are used in a wide variety of applications such as: analysis of vegetation type, monitoring of the forest conditions (biomass, deforestation, changes with seasonal cycles), monitoring of coastline and glaciers (glaciers erosion), monitoring of the environment disaster (forest fires, oil spill, flooding) and so on [1]. While the high dimensionality of hyperspectral data is very advantageous for the image analysis, the on-board storage space and the transmission bandwidth are limited resources. For this reason, it is necessary to set a proper system of on-board data compression before the transmission to the Ground Station. Usually, hyperspectral images show high spatial and spectral correlation among neighbor pixels and among bands. These properties are exploited by compression algorithms that remove the existing redundancy and encode information that is otherwise non-compressible. Many methods are focused on the spatial redundancy and are divided into methods based on predictors [2] and methods based on transforms. Methods based on transform use DWT (Discrete Wavelet Transform) or DCT (Discrete Cosine Transform) [3]. About the spectral redundancy, the proposed algorithms exploit the principle of the PCA (Principal Component Analysis) [4,5]; this approach allows the aggregation of the whole information taken by a remote sensing image in a few uncorrelated bands that are linear combination of the captured ones.

In recent years, there has been a tremendous growth of programmable graphics hardware, both in terms of performance and functionality. Graphics Processing Units (GPUs) have allowed their usage for general–purpose computation, making possible a substantial acceleration of algorithms and taking advantage of their parallel multiprocessors [6]. For many compression algorithms, it has been possible to achieve significant reduction in processing time when parallelized and executed on a GPU [7,8].

The purpose of the paper is to propose a method to perform on-board data compression for hyperspectral remote sensed images. The novelty of the method is to resort to the combination of three different factors: algorithm, implementation, and strategy. The adopted strategy is to perform a fine compression for a category of land cover types that are of interest for the user, leaving with less bits per sample of the remaining ‘background’. The purpose is to perform an effective compression that produces less distortion for the class target of interest. For this reason, an onboard classification of the acquired image is proposed. For this aim, band reduction and spatial reduction are achieved by using Principal Component Analysis and 2D Wavelet, respectively. Finally, the third key element is an efficient implementation on GPU that allows many operations to be parallelized and done efficiently from computational and power consumption point of view.

To summarize, the proposed framework recognizes specific regions of interest (ROIs) in the image using a somehow commanded query (generated by the on-ground control station) and by the means of a computationally efficient clustering algorithm; then, it makes a compression of the image based on the user’s specific request (e.g., events detection or specific land cover type reconstruction with less distortion). All of the steps of the algorithm (K-means clustering, extraction of the signature, labels assignment, PCA, 2D-DWT and quantization) are optimized firstly on a CPU; then, many steps are parallelized on GPU.

The rest of paper is organized as follows: the related works are in Section 2, the adopted framework is presented in Section 3, the results of the experiments are shown in Section 4, discussion and relation with previous studies is in Section 5, and, finally, the conclusions are reported in Section 6.

## 2. Related Works 

A number of works have been produced in the recent years about the use of GPU to support hyperspectral remote sensing image processing, typically target classification or spectral feature extraction.

More in detail, GPU seems to be used especially to support on-ground operations such as: pixel detection or classification and spectral signature extraction. In [9], a weighted spatial-spectral kernel detection is achieved by reconstructing the central pixel using the spatial neighborhood information. Efficient implementation is obtained by using GPU. In [10], the similarity between spectra is measured to discriminate materials and evaluate the performance of parameter-selection procedures. The Ensemble Empirical Model Decomposition is used in this case and spectral features are effectively extracted. In [11], a spectral unmixing algorithm is implemented onto high-performance computing architectures with GPU. In [12], instead, the spectral compressive acquisition method is applied to perform dimensionality reduction based on random projections. A parallel implementation using GPU is then described. More similar to the architecture proposed on this paper (hybrid GPU–CPU), is the paper in [13], which proposes a hybrid architecture to separate different spectrally pure endmembers and find the spectral signature in near-real time.

On the other hand, there is a number of works related to efficient onboard operations. Onboard operations especially aiming at producing efficient data compression, regardless of the image content or without comparison with available metadata (an example: an approximate classification by means of on-board extracted spectral features). In [14], the authors propose maximizing image data transmission efficiency for large volume and high-speed data downlink capacity, by using a fast and lossless image compression system, which is a hierarchical predictive coding method with resolution scaling. In [15], a flexible framework for lossless and lossy onboard compression of multispectral and hyperspectral images is proposed instead. This algorithm is able to control the signal-to-noise-ratio or the rate.

To the author’s knowledge, however, no one has yet proposed an algorithm that puts together both the previous aspects, i.e., (i) an efficient GPU implementation, to support onboard operation (efficiency involves both computational and power-consumption point of view); and (ii) on-board compression that supports recognition of specific targets. The onboard compression proposed in this paper does not ground on particular on-the-edge compression methods found in the recent literature since the goal is in implementing a simple and efficient method that can be easily parallelized and on-board processed, rather than trying to reach the compression ratio limit. The main difference, with a previous work [16], lies in the goal of the work. In [16], there is more attention on the compression efficiency vs. the distortion rate and there is no parallel implementation over a GPU. Here, we focus the attention on the parallel implementation, once the performance in terms of efficiency (bit rate) and accuracy (distortion) have been established.

## 3. ROI-Based on Board Compression 

The work has been carried out in two steps. In the first step, it is supposed that the system is able to recognize specific regions of interest such as particular topographic classes, specific events like disaster or specific limited areas, according to external command based on a specific query from the on-board software. This nontrivial task is reached by applying a clustering algorithm to the image aimed at isolating the classes or regions of interest. The clustering resorts to a simple method, well-known in literature (not to overload the on-board hardware), such as the K-means algorithm. However, an automatic estimation of some critical parameters is applied for a more efficient on-board implementation. The second step consists of the compression and encoding of the ROIs identified in the first step. The aim of these operations is to encode the ROIs with a higher bit rate since they represent the area of interest for the user, while the not-ROIs are encoded using a lower bit rate. To reduce the spectral and spatial redundancy present in the hyperspectral image, PCA and 2D-DWT are used, respectively. Finally, an arithmetic encoder (more efficient in a parallel implementation) generates the output bits. To reduce the processing time, some operations are parallelized on GPU, as detailed in Section 3.4.

### 3.1. ROI Detection

#### 3.1.1. The Clustering Algorithm

Unsupervised classification methods are more suitable for automatic segmentation and identification of ROIs in hyperspectral images, since supervised classification cannot be applied, as the labels (in this case, the topographic classes) are not known in advance. Clustering algorithms identify significant patterns without knowledge of the labels and do not need training phases. For these reasons, clustering methods can bring significant benefits:

- It is not necessary to employ considerable resources to learn features of large sets of images to identify ROIs, making the application general purpose;
- By using the intrinsic characteristics of the image, the computational time is reduced, making possible a real-time automatic detection of ROIs;
- The characteristics of some patterns may change over time, but a clustering algorithm applied on single images circumvent this problem.

An efficient and computationally cheap algorithm is K-means [17]. K-means, through an iterative procedure, partitions the samples by minimizing the sum of squares of distances between the centroid of each partition and all the other points in the set. A great advantage offered by this process is the high convergence speed, while the identification of the optimal parameter K represents the critical step.

In our implementation, the pixel spectral signature (i.e., the reflectance emitted by the same topographic element through the acquired bandwidths) has been chosen to make the clustering. In principle, an optimal number of clusters should resemble a rough topographic classification of the image. To select the optimal number of clusters, an automated method that exploits the statistical characteristics of the image [18] has been used. This method exploits the three-dimensional co-occurrence matrices of the hyperspectral cube [19] to estimate the optimal value of K.

For a two-dimensional image quantized to an *n* level of gray, the co-occurrence matrix (GLCM) describes the spatial relationship among gray level values and its consists of an n×n array obtained counting the number of times, given that a couple of grey levels are present in the image for two pixels separated by a specific offset. The offset is specified by a displacement vector d=(dx,dy), where dx represents the offset in the x-direction and dy the offset in the y-direction. Each entry (i,j) in the matrix represents the total number of times that the pixel with value i occurred in the specified spatial relationship to a pixel with value j. The matrix then provides information about the different combinations of pixel gray levels existing in an image. Formally, for an image I of size P×Q, the GLCM C is defined as:

(1) $$C\left(i,j\right)=\sum_{i=1}^{P}\sum_{j=1}^{Q}\left\{\begin{array}{c} 1\;,if\;I\left(x,y\right)=i\wedge I\left(x+dx,y+dy\right)=j\\ 0,\;otherwise \end{array}\right\}.$$

**Figure 1:**
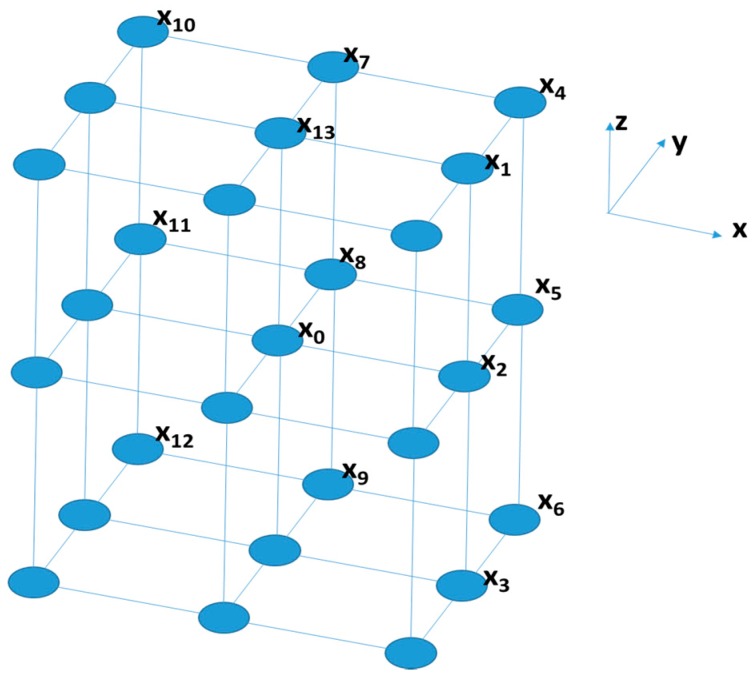
Pixel of interest $X_{0}$ (in the center) and the adjacent pixels in the 13 allowed directions.

In hyperspectral images, it may not be appropriate to analyze independently the individual spectral bands. To extract both spatial and spectral information, co-occurrence matrices are adapted for volumetric data (3D GLCM). These matrices are able to capture the spatial dependence of gray-level value across spectral bands. A 3D GLCM is defined by specifying a displacement vector d=(dx,dy,dz), where dx and dy are the same as described for 2D matrices, and dz represents the offset distance along the spectral axis of the hyperspectral image. However, while for a 2D GLCM only four displacement directions are possible with an offset of 1, in the three-dimensional case, there are 26 admissible co-occurrence directions and only 13 of them differ from each other, considering the symmetries. The 13 directions and their corresponding displacement vectors are reported in Table 1, where D is the offset distance between the pixel of interest and its neighbors, θ is measured in the XY plane in the positive x direction, and Φ is the angle between the vector, which identifies the pixel along the spectral direction and the XY plane. A graphical representation of the pixel of interest $X_{0}$ and the 13 adjacent pixels $X_{i},\;i=1,\ldots .13$ in the directions specified in the Table 1 is shown in Figure 1.

In the implemented algorithm, an offset distance D=1 between adjacent pixels is considered, obtaining 13 3D co-occurrence matrices for the hyperspectral cube. For each matrix, the main diagonal represents the set of pixel pairs with no gray level difference. It has been shown [16,19] that these diagonals give information on clusters of pixels. For each matrix, the following operations are performed:

- Extraction of the main diagonal;
- Computation of the histogram of the values of the main diagonal;
- Detection of the local maximum of the histogram.

The gray value corresponding to the local maximum is an indication of the number of clusters present in the considered co-occurrence direction. The total number of clusters of the hyperspectral image is estimated by the maximum value among the 13 local maxima.

#### 3.1.2. Automatic Extraction of Signatures and ROI Labeling Process

Let us suppose that a set of Top of Atmosphere (TOA) reflectance of the most interesting and common classes of land cover types have been pre-loaded in the on-board memory (possibly, such profiles can be refined through successive acquisitions).

After the identification of the clusters, the K spectral signatures represented by the centroid vectors are computed and saved in the on-board memory. Such spectral signatures are the radiance of representative land cover types and must be corrected for the atmosphere effects to get the real TOA reflectance. The correction of atmospheric effects is a quite automated operation (https://landsat.usgs.gov/using-usgs-landsat-8-product), but the automated correction of atmosphere impact has rarely been tackled in literature (sun and sky glint on water surface, [20]), [21], and, in principle, it requires further processing. The authors have not faced with this aspect of the problem (computational effort and time) and they postpone such analysis to future study.

To relate the spectral signature of each cluster with the most likely pre-loaded reflectance (said label), the highest correlation coefficient among them is used [22]. In relation to the user query, i.e., to the event or specific class of interest to detect, the image is segmented distinguishing ROI from not-ROI areas (a minimum size of the segment is a pre-defined parameter).

### 3.2. ROI and Not-ROI Compression and Encoding

To reduce the data volume that would be generated by a hyperspectral image, the high spectral and spatial correlation are exploited. The spectral redundancy is reduced by applying Principal Component Analysis (PCA), which transforms a set of correlated data into linearly uncorrelated variables, known as Principal Components. PCA is used to decompose a multivariate dataset into a set of successive orthogonal components that explain the maximum amount of the variance. The complexity reduction is achieved by keeping only the first principal components, up to a given percentage (typically a number between 90% and 99%) of the cumulative explained variance.

To reduce the spatial redundancy, Discrete Wavelet Transform (DWT) is used instead. The DWT is a method for multiscale (i.e., multiresolution) signal or image analysis and can also be adopted for compression, if just a few coefficients are retained after transform. After the application of a prototype function and its scaled versions, the image is successively decomposed into a set of lower resolution (approximation coefficients) and details, which may be viewed as successive low-pass and high-pass filtered versions of the original image. For each ROI (or not-ROI) and band, the 2D-DWT is performed. For each band, said (A, B), the image sizes (rows, columns), after the 2D-DWT operation, four sub-band (C, H, D, V) images, each with A/2 rows and B/2 columns, are obtained. The sub-band C has the highest energy compared to the other sub-bands, since it corresponds to the low-pass horizontal and vertical coefficients of the wavelet transform. The 2D-DWT of the image g(x,y) of size A × B is:

(2) $$W_{\delta }\left(a,b\right)=\frac{1}{\sqrt{AB}}\sum_{y=0}^{B-1}\sum_{x=0}^{A-1}g\left(x,y\right)\delta_{a,b}\left(x,y\right),$$

(3) $$W_{\vartheta }^{k}=\left(j,a,b\right)\frac{1}{\sqrt{AB}}\sum_{y=0}^{B-1}\sum_{x=0}^{A-1}g\left(x,y\right)\vartheta_{j,a,b}^{k}\left(x,y\right)\;with\;K=H,D,V,$$

(4) $$\delta_{a,b}\left(x,y\right)=\delta \left(x-a\right)\delta \left(x-b\right)=\;\;\sum_{m}h_{\delta }\left(m-2a\right)\sqrt{2}\delta \left(2x-m\right)\times \sum_{n}h_{\delta }\left(n-2b\right)\sqrt{2}\delta \left(2x-n\right),$$

(5) $$\vartheta_{j,a,b}^{H}=2^{\frac{j}{2}}\vartheta^{H}\left(2^{j}x-a,2^{j}x-b\right)=\;2^{\frac{j}{2}}\sum_{m}h_{\delta }\left(m-2a\right)\sqrt{2}\delta \left(2^{j+1}x-m\right)\times \sum_{n}h_{\delta }\left(n-2b\right)\sqrt{2}\delta \left(2^{j+1}x-n\right),$$

where δ is a scaling function, $W_{\delta }\left(a,b\right)$ the approximation coefficient of the function g(x,y), $W_{\vartheta }^{k}\left(j,a,b\right)$ the coefficients relative to the horizontal, diagonal and vertical details and $h_{\vartheta }$ and $h_{\delta }$ are the wavelet filters. For our purposes, we have used a biorthogonal wavelet filter [23,24] after the verification that this wavelet is able to minimize the distortion in the final images and after the approximation achieved by using just the low-pass coefficients.

### 3.3. Quantization and Encoding

The DWT implementation is followed by an operation of uniform scalar dead-zone quantization (USDZQ) [25], in which each component C, in relation to the label, is quantized with a different number of bits, which is anyway smaller than that of the original acquisition. The quantization indices are expressed as:

(6) $$q\left[i\right]=sign\;\left(C\left[i\right]\right)\lfloor \frac{C\left[i\right]}{\Delta }\rfloor ,$$

where *C*[*i*] denotes the sample of sub-band *C* (i.e., the approximation coefficient after the wavelet transform) and Δ is the quantization step. The ROI areas are quantized with a number of bits per sample larger than the not-ROI areas.

Finally, an entropy encoder is applied. Entropy coding is a variable-length encoding that reduces the volume of data to be transmitted by removing, in principle, all of the redundance. This way, the average number of bits per sample approaches the theoretical limit, i.e., the entropy source:

(7) $$H\left(P\right)=-\overset{n}{\underset{k=1}{\sum }}p\left(k\right)log_{2}p\left(k\right),$$

where *p*(*k*) is the probability of emission of symbol $x_{k}$. In this case, the Arithmetic encoding, a symbolwise recursive encoding algorithm, is used [26,27]. Arithmetic encoding evaluates the probability with which each symbol appears and optimizes the length of the required code. In order, the idea is to:

- break the source message into symbols, where the symbol is some logical grouping of characters;
- associate each distinct symbol with a interval of the unit [0…..1);
- tighten the unit interval by an amount determined by the interval associated with each symbol in the message;
- represent the final interval by choosing some fraction within it.

The on-ground decoding operations are performed by reversing the on-board steps: entropy decoding, inverse 2D-DWT and reconstruction from components of PCA. Some additional metadata are packetized in the data sent on ground. Among these metadata are: the block size, the coordinates of identified ROIs (or the timestamp of the image acquisition), the PCA vector or means, the number of bits per pixels for ROI and not-ROI regions and the selected labels for the clusters.

### 3.4. Implementation on GPU

On-board processing operations are usually performed in critical conditions: shortage of computational resources and of power, together with requirements to process the dataset in a useful amount of time for download or for the successive acquisition, make any post-processing on datasets a challenge. For this reason, reduction of processing time and/or number of operations is highly beneficial. For this aim, GPU has been adopted, since it has low power consumption, light weight and high computing capability [28,29].

**Figure 2:**
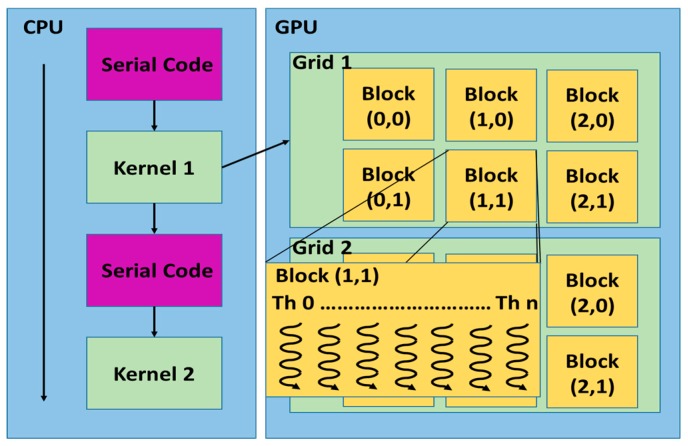
Grid, blocks and thread in a Graphic Processor Unit (GPU). (Figure 2 and Figure 3 redrawn from the “NVIDIA CUDA C Programming Guide” v. 4.2, pag. 9. NVIDIA Corp., Santa Clara, CA, USA).

The GPU architecture can be seen as a set of Multiprocessors (MP) characterized by single instruction multiple-data. Each processor executes the same instruction in each clock cycle and it has access to a local shared memory while the MPs have access to the global memory of the GPU device. The code executing in parallel on GPU is called kernel. As shown in Figure 2, a kernel is executed as a grid of blocks of threads.

**Figure 3:**
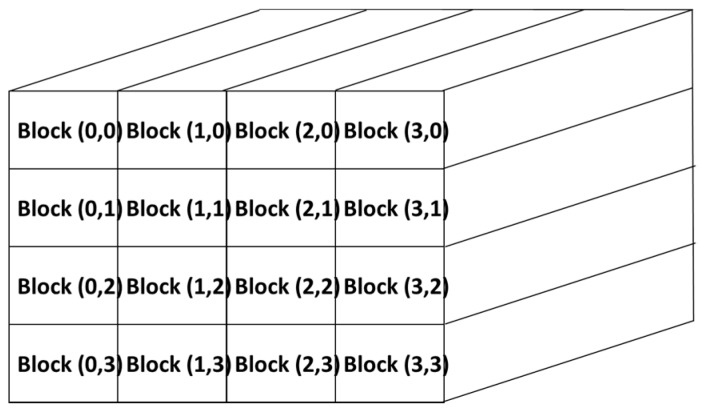
Partition of the hyperspectral image into fixed-size blocks.

In the proposed method, the hyperspectral image is mapped into the GPU memory. The number and the block size are function of the size of the acquired image. After the data has been copied into the global memory of the GPU device, the entire hyperspectral image, seen as a 3D grid, is divided in fixed-size blocks (Figure 3).

For each block, each core executes the same functions (in a parallel way): K-means, extraction signature, label assignment (“ROI” or “not-ROI”), PCA, 2D-DWT, USDZQ. The coordinates and labels related to each block are stored and sent to the ground as metadata, so to aid the reconstruction software to regenerate the image. The final step, the arithmetic encoding, is performed in the CPU because it is sequential and cannot be parallelized. Indeed, the entropy encoders are mainly sequential and there is a strong data dependency. The parallelization has been carried out using NVIDIA GeForce GTX 750 Ti (NVIDIA Corp., Santa Clara, CA, USA) with 2 GB of random access memory (RAM). The main property of the hardware are in [30].

**Figure 4:**
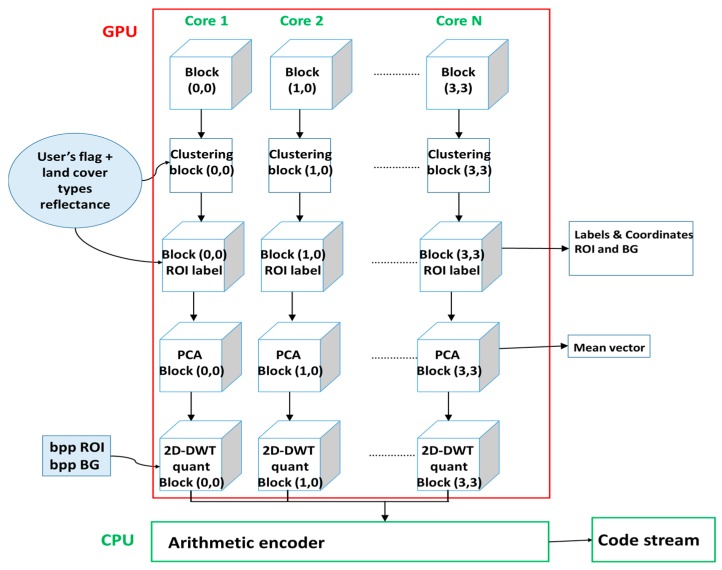
Block diagram of the implemented algorithm.

The implemented algorithm is finally shown in Figure 4.

## 4. Results

Experimental results have been achieved by using an image taken from an airborne hyperspectral sensor. We formulated a possible query reproducing a reasonable request of a final user of the product. The query concerns the selection of image sub-blocks that include “city” land cover types (cement, asphalt), to be distinguished from background (any other land cover type such as sea or open vegetation). Such ROI are compressed using more bits than in the remaining background. As a measure of performance, the processing time has been estimated (CPU implementation vs. GPU-parallelized implementation) for each single processing step. Then, the size of the block has been changed to estimate an optimal memory management within the GPU. Finally, since the arithmetic encoding affects the final accuracy in the reconstruction, different bits per pixel value have been tested to analyze the increase of processing time. The reconstruction accuracy and the generated volume of data, although estimated in a previous work [16], has been reported again for a complete discussion about the trade-off between distortion, generated volume and processing time.

### 4.1. Dataset Description

**Figure 5:**
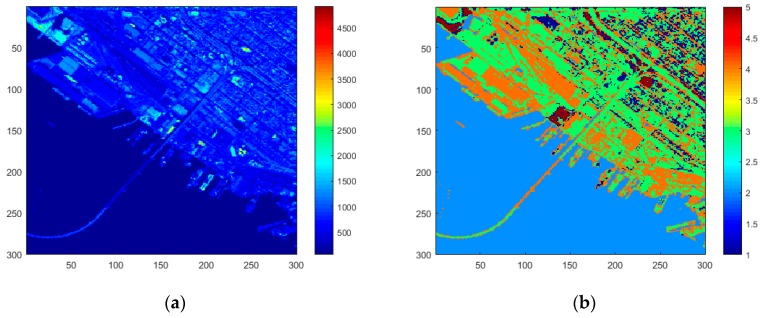
(**a**) Input image (band = 50), (**b**) results after clustering algorithm.

A real dataset has been used to test the algorithm in terms of efficiency in recognizing the target and in terms of computational effort. The hyperspectral image has been captured by the AVIRIS (Airborne Visible/Infrared Imaging Spectrometer) sensor [31]. The 300 × 300 AVIRIS hyperspectral image has 224 continuous spectral channels with wavelengths from 400 to 2500 nanometers, and, in each band, the reported value represents the land and sea reflectance. The targets to be detected consist of ships near the harbor area of San Diego (CA, USA) (Figure 5a).

### 4.2. Processing Steps

After the clustering operation described in Section 2, K = 5 different classes have been detected. The clustered image is shown in Figure 5b, where the accuracy in distinguishing sea area from city or from the port facilities can be appreciated. The sea is identified with index 2, the vegetation with index 1 and cement and asphalt with 3 and 4, respectively. Index 5 identifies another land cover type, perhaps bare soil. It is noteworthy that clustering of an image can be executed just one time, during the first monitoring of a given area, according to the capability of the on-board system to store information about specific areas.

To relate the clusters label to specific land cover types, we suppose that the reflectance of to sea, vegetation, cement and asphalt have been pre-loaded on board and can be used to make assignment. In fact, the five classes, identified with the K-means algorithm, are discriminated with the on-board signatures (saved as array of 224 elements) through the evaluation of the correlation coefficient. Each block is labeled as ROI if the pixels belonging to the “sea class” occupy from 10% to 90% of the total block and those belonging to “cement” and “asphalt” occupy from 2% to 40% (these values have been optimized after some experimental trials); otherwise, the label “not-ROI” is assigned to the block.

PCA is then implemented to reduce the spectral bands. In this case, the principal components representing the 99.9% of the explained variance have been retained. They resulted in 18 principal components, allowing the reduction of the data volume to 8% (i.e., 18/224 bands) of the initial one.

### 4.3. Performance Results

The performance of the algorithm has been already presented in [16] and is shortly summarized here. The algorithm has been evaluated in terms of accuracy in reconstruction and data volume generated. The accuracy has been measured using the distortion index (DI) that is the inverse of the mean square error between the reconstructed and the original image. In order to have a fair comparison, the DI has been calculated only in the region of interest (ROI), instead of using the entire image (ROI and background). The reason of this choice is that the comparison would be otherwise unfavorable to the proposed framework and not consistent with the purpose of the algorithm, i.e., the transmission of a reduced data volume to the ground.

**Figure 6:**
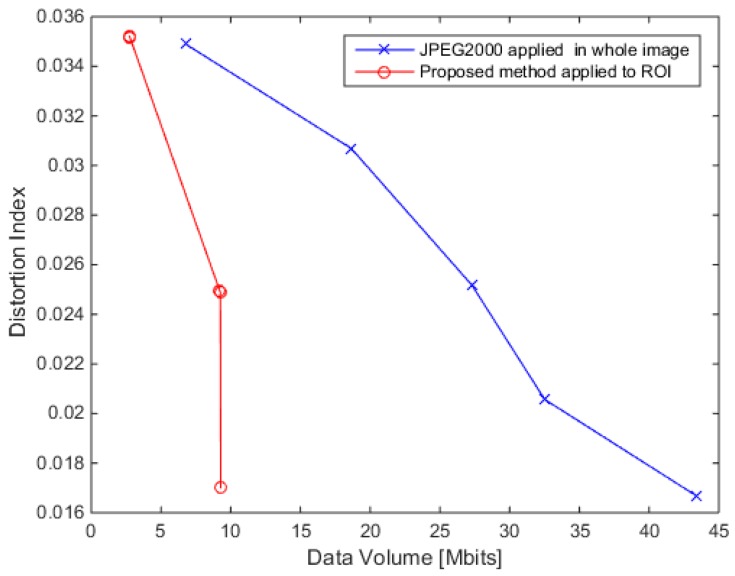
Distortion Index as a function of the data volume for the investigated image in Figure 5a.

In Figure 6, the algorithm performance has been compared with the JPEG2000 compression method for different compression depths (i.e., number of bit per pixels for the ROI or for the overall image in the JPEG2000 case). The algorithm has superior performance in the ROI region (at equal distortion, the generated volume is lower). The drawback is clearly the high compression quality available only for the region of interest and not for the entire image.

### 4.4. Implementation Results

After the optimization of all functions on CPU, the implementation on GPU has been performed. The results have been achieved using a computer mounting an Intel i7 4790 CPU processor @ 3.60 GHz with 16 GB of RAM and 64-bit architecture and, as GPU, a NVIDIA GeForce GTX 750 Ti. This GPU allows a maximum dimensionality of grid of thread equal to 3, maximum *x*-dimensionality of grid of threads block equal to $2^{31}$−1 and maximum *y*- or *z*-dimension equal to 65,535. The hyperspectral data have been copied in the GPU global memory.

#### 4.4.1. Comparison of CPU and GPU Implementation

**Figure 7:**
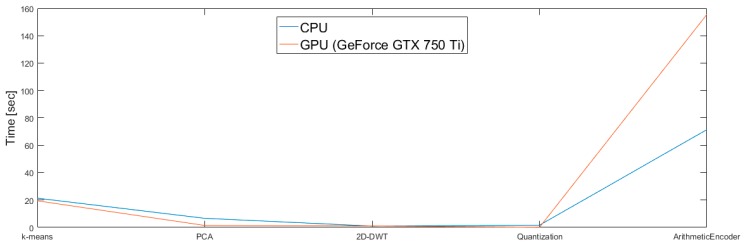
Processing times for each processing step, separately for CPU or GPU implementation.

Figure 7 shows the contribution to the total processing time of each operation (clustering, PCA, 2D-DWT, quantization and encoding). As can be seen, K-means, PCA, DWT and quantization present an advantage in terms of processing time on GPU rather than on CPU. As expected, instead, the arithmetic encoding is more convenient on CPU, proving that this operation is not parallelizable.

Putting together the processing operations, Table 2 presents the total execution time on CPU, GPU and, as optimal configuration, the implementation of all the steps on GPU but for the arithmetic encoding (executed on CPU because of its intrinsic sequential nature). The optimal configuration (GPU + CPU) allows a speedup of 3.21 times the processing time compared to the CPU implementation.

The different implementations on the CPU and on GPU + CPU have been then tested to verify that the same result is achieved in both the cases. The serial implementation on CPU and the proposed parallel GPU + CPU method provided the same results up to the machine precision. The comparison has estimated by computing the means square error of the two implementations:

$$\mathrm{mean}(|{\mathrm{ImageReconstructed}}_{\mathrm{CPU}}-{\mathrm{ImageReconstructed}}_{\mathrm{GPU}\;+\;\mathrm{CPU}}{|}^{2})$$

and has been found to be less than 10^−6^.

#### 4.4.2. Results for GPU Parallel Implementation 

**Figure 8:**
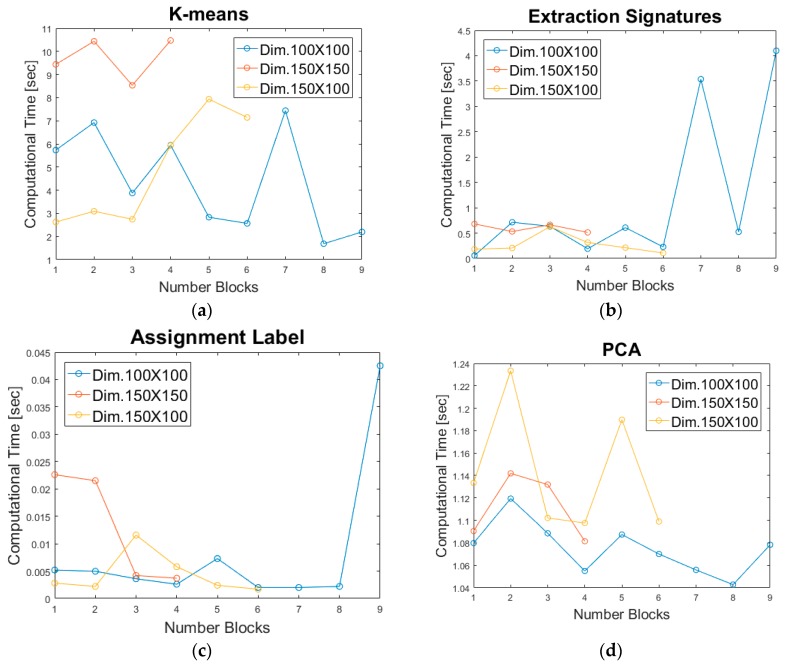
Processing time for each block, for different block size. (**a**) K-means processing time; (**b**) extraction signatures processing time; (**c**) assignment label processing time; (**d**) PCA processing time.

The hyperspectral image has been tested in parallel on multiple cores for different block sizes. Data have been divided in blocks of size 100 × 100 × 224, 150 × 150 × 224, 150 × 100 × 224 and processed in four cores. In the proposed processing strategy, each core processes a separate block. The block size clearly conditions the output of the single processing steps, as evidenced by Figure 8. In principle, more blocks mean that the process can be highly parallelized, but this does not mean always a reduction of the total processing time because of the request of more parallel resources. Figure 8a–d show the processing times of all functions processed by the GPU for each individual block, compared for the different sizes. In relation to the block size, the blocks are processed in parallel: nine blocks in the first case (blue line), four blocks in the second case (red line) and six blocks in the last case (yellow line). Figures show that the number of parallel processes (core) conditions the processing time. 2D-DWT and USDZQ operations (carried out in parallel on the principal components) are not shown in Figure 8 since such processes take a few milliseconds.

The block size of the ROI is a function of the amount of shared memory, the image size and the number of available kernels that can be launched in parallel. The smaller the size of an ROI, the higher the degree of parallelization, but the higher the parallel resources that were needed. For three block cases and using an 8-bit per pixel quantization for the ROI, the GPU + CPU execution times are reported in Table 3.

#### 4.4.3. Processing Time for Different Accuracy 

The number of bits per pixel assigned by the user affects only the arithmetic coding operation. In Table 4, not-ROI areas have been encoded with 2 bpp, while ROIs have been encoded with a variable number of bits per pixel. As can be seen, the number of bits per pixel used in the arithmetic encoding affects the processing time and, of course, also the final quality of image (less bits per pixel in the ROI produces a less accurate reconstruction).

The final number of bits per pixel to assign to the ROI is a function of the specific target or event to detect and of the accuracy with which the target must be represented. Estimation of this number in a general case goes beyond the scope of this paper.

## 5. Discussion

Real operations of a multispectral acquisition camera may hinder the actual application of the algorithm. Some factors, in fact, have not been included in the discussion since they are beyond the purpose of the paper. A first issue is the conversion of the acquired image (which is the ground radiance) into reflectance, in order to have a fair comparison with a database of spectral signatures, in order to identify the classes (partly discussed in Section 3.1.2). Another aspect is the computational effort that translates into an amount of energy consumption. Current GPUs are energy-efficient systems, but the number of floating point operations per second may be a problem even for energy-saving hardware (about 800 nW per multiplication is estimated for on-board systems [32,33]). Moreover, the capability of the algorithm to assign reliable labels to land cover classes is limited by the nature and the amount of spectral signature that the on-board storage system may keep. A more refined algorithm could include an orbit-sensitive detector that may load specific classes according to the position of the satellite (example: sea and ice classes at high latitudes, vegetation, urban, rock and soil at mid latitude and so on). Moreover, a cloud detector algorithm should be always active to discard the images with too high a cloud coverage. Finally, a third aspect involves the operation time to conduct processing. We have had no opportunity to test faster GPUs; however, it is realistic that a powerful GPU mounted on board of the next-generation satellites may overcome the current time processing estimate or work in parallel on different images serially acquired, so to make a single download at the ground station. Another possibility is to change some processing steps with others that are more efficient. In literature, some algorithms for efficient image segmentation have been proposed. Among these, saliency detection seems quite promising [34], although it requires a high computational burden since detection requires the analysis of the image texture. The authors have not investigated in this direction.

A final aspect includes the refinement of the algorithm parameters. The image block size is clearly the most important factor for the processing time in a parallel implementation and it is a function of the nature of the image but also of the GPU processing capabilities. It should be tested with on-ground standard images before real operations, in order to optimize both the representation accuracy and the processing time. Another issue involving the accuracy in reconstruction is the number of principal components to retain after PCA and the depth of the wavelet transform. Again, such numbers are image-dependent and thoughtful on-ground tests should be carried out to reduce the distortion in reconstruction. An alternative to the space variability of the images may be, again, the possibility to tune such parameters on board by an automated algorithm (orbit dependent) or by on-ground tele-command.

## 6. Conclusions

The paper proposes a method to reduce the on-board data volume produced by a hyperspectral acquisition, keeping, at the same time, a high accuracy in reconstruction of specific target (i.e., land cover class or event) on ROI. This system works mainly using two elements: (i) a clustering algorithm that automatically segments the image; and (ii) a faster ROI-based compression algorithm. These features make the algorithm suitable for real-time (or near real-time) detection of events and recognition of specific land cover classes.

The parallel computing capabilities of GPU allows the reduction of the processing time, but future improvement in both accuracy and time of execution must be investigated. This includes: (i) further tests in different scenarios to increment the sensitivity of the system in recognition of specific events or classes (example: fast identification of oil spills or forest fires); (ii) implementation of the algorithm on FPGA (Field-Programmable Gate Array) and comparison with GPU performances; and (iii) automated selection of algorithm inner parameters such as the number of bits per pixel for ROI and background.

## Figures and Tables

**Table 1:** Displacement vectors and directions of co-occurrence matrices for volumetric data. The first two values represent the displacement in the same plane, and the third one represents the displacement through different spectral images. D is an arbitrary integer value.

Displacement Vectors	Directions (θ, Φ)
(D,0,D)	(0°,45°)
(D,0,0)	(0°,90°)
(D,0,−D)	(0°,135°)
(D,D,D)	(45°,45°)
(D,D,0)	(45°,90°)
(D,D,−D)	(45°,135°)
(0,D,D)	(90°,45°)
(0,D,0)	(90°,90°)
(0,D,−D)	(90°,135°)
(−D,D,D)	(135°,45°)
(−D,D,0)	(135°,90°)
(−D,D,−D)	(135°,135°)
(0,0,D)	(-,0°)

**Table 2:** Processing times of the entire algorithm on the CPU and the GPU and in the mixed case.

Size Block (pixel)	CPU Time (s)	GPU Time (s)	GPU + CPU Time (s)
100 × 100	60.8227	77.3068	18.9352

**Table 3:** Total algorithm processing time for different size block (elaborated in parallel by four cores).

Size Block/Number Blocks (pixel)	Total Execution Time (s)
100 × 100/9 blocks	50.858
150 × 100/6 blocks	46.4282
150 × 150/4 blocks	40.4222

**Table 4:** Processing time of arithmetic encoding for different bits per pixel for the ROI.

AritEnco 8bpp Time (s)	AritEnco 4bpp Time (s)	AritEnco 2bpp Time (s)
15.1296	6.7934	2.8123
